# Lectures, Illustrative of Various Subjects in Pathology and Surgery

**Published:** 1846-07

**Authors:** 


### Art. XIII.

*Lectures, illustrative of various Subjects in Pathology and Surgery.* By Sir Benjamin Brodie, Bart., f.r.s.—*London,* 1846. 8vo, pp. 412.

The pen of Sir Benjamin is at all times practical; and, as such, its labours are well appreciated by the profession. Whatever appears under his name and sanction is sure of attentive perusal, by all who care to inform themselves in the current literature of the day; and few, if any, will rise from such perusal without being very sensible of having obtained no inconsiderable amount of profit and instruction.

The Lectures now before us have been delivered at various times; and, through the journals of the day, the majority have already been made public, but in such a detached manner as to render their previous printed existence of but little avail for the purposes of consultation and reference. We rejoice to see them now collected by the author himself, and by him given forth in a solid, suitable, and satisfactory form. For more than one obvious reason, the contents of the volume are unsuited to ordinary criticism; and we believe that we shall best discharge our duty by abstaining from all other notice of them than what is necessary to convey to such of our readers as may have not yet perused the volume itself, a digest of the practical bearing of the prelections.

Lectures I and II.—These have much to do with the ethics of the profession, and are chiefly addressed to its youngest members—students entering upon their studies; the first comprehending "the studies required for the medical profession;"* the second, treating of "the duties and conduct of medical students and practitioners." Both are admirable. Were we responsible for a text-book, or system of surgery, we should be sorely tempted to perpetrate an open act of piracy, and print them *in extenso* as the most suitable of all commencements of a new edition. Of these we can give no abstract; they must be read to be felt and understood. We cannot, however, refrain from giving a specimen, though small, of each.

In the first Lecture there occurs the following paragraph, the moral of which cannot be too strongly urged upon the rising generation. Every one acquainted with medical tuition must be familiar with very many painful examples of the sad results of the neglect of its truthful axiom.

"It is not uncommon for medical students, any more than it is for other students, to engage at first with zeal in their pursuits; then, as these lose the charm of novelty, to become careless and indifferent, and at last, when their education is drawing to a close, and it becomes a question how far they are qualified to undergo the required examinations, to endeavour to make up for the time which has been misspent and wasted, by excessive labour, such as is incompatible with sufficient physical repose and mental relaxation. But it is not in this way that great things are to be accomplished, either in our profession or in any other. Habits of attention which are once lost are not easily regained; and no durable impressions are made upon a mind which is exercised beyond its powers. The slow but persevering tortoise reached the goal before the hare, who was over confident of

* Addressed to the Students of the Medical School of St. George's Hospital; the one in 1838, the other in 1843.

the speed which she could exercise if she were required to do so; and this fable, which we were taught in the nursery, conveys a moral lesson which the philosopher need not be ashamed to learn." (pp. 5-6.)

In the second lecture there is much sound advice for old as well as young. What more true than this?

The self-sufficient, who do not keep before their eyes an ideal standard of perfection, who compare themselves only with those who are below them, will have an advantage with inexperienced and superficial observers; but I must say that I have never known any one to do any real good in the world, or obtain ultimately a bright reputation for himself, who did not begin life with a certain portion of humility. The greatest men are humble. Humility leads to the highest distinction, for it leads to self-improvement. It is the only foundation of a just self-confidence. Study your own characters; endeavour to learn and to supply your own deficiencies; never assume to yourselves qualities which you do not possess; combine all this with energy and activity, and you cannot predicate of yourselves, nor can others predicate of you, at what point you may arrive at last." (pp. 34-5.)

Or this?

"There is no profession in which it is more essential that those engaged in it should cultivate the talent of observing, thinking and reasoning for themselves, than it is in ours. The best part of every man's knowledge is that which he has acquired for himself, and which he can only to a limited extent communicate to others. You will spend your lives in endeavouring to add to your stores of information; you will, from day to day, obtain a clearer and deeper insight into the phenomena of disease; you will die at last, and three fourths of your knowledge will die with you; and then others will run the same course. Our sciences are, indeed, progressive; but how much more rapid would their progress be, if all the knowledge that experience gives could be preserved." (pp. 38-9.)

Of two things let no member of our profession prove oblivious. First, that his profession both requires him and entitles him to be a gentleman. Second, that there is both a real and a false assumption of "the gentleman" to be found in the world. And if he have any doubt as to which is which, let him turn to page 52 of the present volume and he will then find the following solution of his difficulty.

"It is not he who is fashionable in his dress, expensive in his habits, fond of fine equipages, pushing himself into the society of those who are his superiors in their worldly station, that is entitled to that appellation. It is he who sympathizes with others, and is careful not to hurt their feelings even on trifling occasions; who, in small things as well as in great, observes that simple but comprehensive maxim of our Christian faith, 'Do unto others as you would they should do unto you;' who, in his intercourse with society, assumes nothing which does not belong to him, and yet respects himself; this is the kind of gentleman which a medical practitioner should wish to be." (p. 52.)

Lecture III. This is "*on the effects of strangulation;*" and it, and the following, though now published for the first time, were delivered in the theatre of the Royal College of Surgeons, in London, as long ago as the year 1821. The subject is physiological, but the object thoroughly practical. And it is obviously most important for every practitioner to be well versant in this matter; as, without a knowledge of the physiological department, he will make but a sorry figure in a witness-box, under the fire of a cross-examining barrister; and without familiarity with the required surgical treatment, he will be worse than useless in the professional emergency.

Strangulation may affect the vertebræ, the blood-vessels, the nerves, the muscles, and the trachea. Even in cases of criminal suspension, at least in this country, it is seldom that the bones suffer, either by fracture or displacement. The inner coat of the carotid has been found ruptured. The veins must always be so compressed as to cause accumulation of blood in the vessels of the brain; and sometimes, though rarely, cerebral apoplexy has resulted from this; but "neither vascular congestion, nor sanguineous apoplexy, is the common cause of death from strangulation." Pressure on the nerves of the neck is not likely to be such as to prove altogether fatal to their function, even for a time. Muscles may be ecchymosed and torn; but that is still more trivial. "There can be no doubt that strangulation causes death by closing the trachea, or preventing respiration; and that whatever other effects it produces are of secondary importance to this." May we be permitted to add, that in the execution of criminals, and in cases of strangulation of a like nature, in which there is any considerable "drop" given to the patient, there is more or less *concussion* inflicted on both brain and spinal cord; which not only tends towards the extinction of life at the time, but proves also a serious obstacle to resuscitation subsequently attempted.

The following is the *mode of death*:

"1. The trachea is obstructed, so that air cannot enter the lungs. 2. The blood passing through the lungs does not undergo that change which respiration produces, and which is necessary to life. 3. Dark-coloured blood, which has not been purified by exposure to air, is transmitted to the left side of the heart, and from thence to the brain and other organs. 4. The heart continues to act, circulating dark-coloured blood, but its actions gradually become weaker, and, in the course of a very few minutes, cease altogether." (pp. 60-1.)

The heart is the *ultimum moriens*; and ceases to act, according to our author, not from insufficient stimulus applied to it by the blood, but from impairment of its nervous energy—the result of black blood poisoning the brain. Mr. Beck has recently observed that the cardiac nerves are accompanied "by processes or elongations of the gray material of the nervous system;" and this confirms Sir Benjamin in his belief that these nerves "possess the property of generating nervous influence," and that on their nervous influence the movements of the heart depend.

*The symptoms of strangulation* follow this course: 1. Discoloration of the lips, and other parts; the result of venous congestion, and of the want of oxygenated blood. 2. Involuntary actions of muscles; convulsive; not accompanied with pain, probably, and caused by "contact of the dark-coloured blood with the brain and spinal cord." 3. The diaphragm and intercostal muscles continue attempts to act, respiratorily,—on an average from a minute and a half to two minutes. 4. When the efforts at inspiration cease, "the pulse of the head and arteries is still distinctly to be felt; and the action of the heart continues of sufficient strength to maintain the circulation for two or three minutes longer." 5. The heart having fairly ceased to act, death is fixed; recovery is impossible.

If the ligature be removed from the neck before the contractions of the diaphragm have ceased, air is drawn into the lungs, respiration is restored, the blood is again oxygenated, and the animal rallies. Also, at a further stage, if during the short interval during which the heart continues to act, after the contractions of the diaphragm have ceased, artificial respiration be maintained, circulation and oxygenation are continued, and normal respiration is resumed. But the heart having fairly ceased to act, Sir B. Brodie is decidedly of opinion that nothing can restore the animal—that the heart will not again be brought to act so as to circulate the blood.

*Treatment* resolves itself into simple indications ; it being remembered, however, there are necessarily but few cases in which the surgeon has an opportunity of hopeful interference. 1. If the ligature is removed before the efforts of the diaphragm have ceased, "all that you have to do is to watch the patient carefully ; if natural respiration continue, leave him to himself ; if it cease, supply the want by inflating the lungs artificially." 2. If the efforts of the diaphragm have already ceased, have recourse to artificial respiration without delay. There is no time to lose. In two or three minutes, after the last heave of the chest, the heart's action will have ceased, and then all hope is over. 3. In successful cases, so soon as normal respiration is established, inflation is desisted from. But treatment is not to cease. The patient is not safe. Dark blood has been circulating in the brain ; and symptoms like those of poisoning by a narcotic may exist. Coma may remain. By and by the respiration may cease. Then has arrived a second period at which artificial respiration may be necessary to preserve life. And, in truth, the practitioner may expect to be called upon to inflate the lungs more frequently at this second period than at the first.

As to the *mode of inflating the lungs,* it is obvious that in the hurry and excitement of the emergency, we are not to trust to syringes, bellows, tubes, elastic-gum bottles, or other contrivances which may be constructed very ingeniously and suitably for this express purpose. They are not to be had. And the surgeon is lucky if he can secure a common bellows. In a certain rural and remote district of Scotland, the belated traveller has found that "there is nae wale o' wigs," and wisely secured the first that came to hand. And so here. Secure the bellows. And if that cannot be had, look for a tube of any kind which may be inserted into the nostril ; a large elastic catheter is very suitable ; if this cannot be got, roll up a piece of card into a cylinder, and with this, and one's own lungs, a tolerably efficient substitute for bellows may be put in play. Then the following practical points require attention : 1. Avoid undue forcing of air into the lungs ; otherwise the air-cells may be burst ; air may enter the blood-vessels, and the result is certainly fatal. 2. Inflate at proper intervals ; imitating as clearly as possible the rhythm of natural respiration. 3. There is no necessity for warming or oxygenating the air ; the attempt is just time lost. And it is fortunate that such manœuvres are not essential ; seeing that the surgeon is not likely to be provided with either a pocket stove or a portable gasometer. 4. The upper part of the body is exposed ; so that the movements of the chest may be accurately noted. 5. The inflating tube is introduced into one nostril. There is no necessity for opening the trachea ; that is only required when previous disease has obstructed the larynx. 6. The other nostril and the mouth should not be closed. They are safety-valves, by which over-distension of the lungs is prevented. 7. An assistant presses the box of the larynx against the vertebra, so as to prevent inflation of the stomach. Were this cavity filled, the descent of the diaphragm would be prevented, and no air could enter the lungs. 8. Electricity and galvanism are inferior to the bellows, for they waste time in application. And the following assertion—repeating more broadly the opinion already stated—hurries him who believes it to the best employment of the very few moments he has to spare.

"*If that action of the heart by which the circulation is maintained should cease, as a consequence of the suspension of respiration, it can never be restored. This I positively assert, after having made it the subject of a very careful investigation.* If others have held a different opinion, it is because they have confounded those feeble and irregular contractions of the heart, which may last for a long time, but which mean nothing, with those regular and powerful movements which are necessary to propel the blood through the system." (p. 80.)

In the after-treatment—natural respiration having been restored—it may be necessary to abstract blood, on account of congestion; but this must be done with extreme caution seeing that the powers of life have been brought low by the faulty circulation in the brain. The warm bath is not essential. But the patient should be kept in an atmosphere of a moderately warm temperature, "to compensate for the insufficient generation of animal heat, which is the consequence of the impaired state of the functions of the brain, whether arising from the influence of a narcotic poison, or from another cause."

LECTURE IV, treats of *drowning*. The lamprey and benito can often vary their existence, pleasantly, by keeping their heads in air; but man cannot follow their example by immersing his head in water with impunity. Death takes place, as if by strangulation; in both cases "the want of due oxygenation or decarbonization of the blood being the sole cause of the animal's destruction." After immersion, a deep expiration takes place, by which bubbles of air are expelled from the lungs. Then comes an ineffectual effort to inspire; but water does not enter, instead of air; spasm of the muscles of the larynx seeming to prevent this. The attempts to breathe are repeated several times, and after each attempt a small quantity of air is expelled from the mouth and nostrils, until the air-cells of the lungs are almost completely emptied. Then insensibility occurs, and convulsive actions of the muscles mark the instant when the brain begins to suffer from the influx of the dark-coloured blood. Soon all motion ceases; save in the thorax, where the heart may be felt yet feebly pulsating. Perhaps some further ineffectual efforts at respiration are resumed, and then all is still. The interval between cessation of respiratory effort and cessation of the heart's action is brief in the case of strangulation; but it is still more brief in drowning. And the whole succession of events, in the latter case, succeeding rapidly, are complete within a very few minutes. Our author is firmly of opinion that in cases of complete and uninterrupted submersion, life has never been retained after more than five or six minutes have elapsed under water. All stories to the contrary he holds to be apocryphal; and, we believe, most justly.

"The cases which have been reported to the Royal Humane Society of drowned persons who have been restored to life, when taken up cold and breathless after an immersion of half an hour, show that it is not travellers alone that are guilty of the vices of exaggeration and invention. We are compelled to regard these as mere extravagant fables, not more authentic, though certainly less poetical and elegant, than those of nymphs and mermaids, who reside in grottoes beneath the waves of the sea, or than those Arabian fictions, which have astonished our youthful imaginations with the history of submarine nations, whose princes dwell in palaces of crystal at the bottom of the ocean." (p. 90.)

The time during which divers, professed and accomplished divers, are able to remain under water, probably never exceeds two minutes; although it may *seem* to be much longer, and the exaggeration of the time of submersion, by a bystander, in the case of either drowning or diving, may very readily be imgained to take place as it were involuntarily, without any intention to deceive; the observer being himself deceived as to the lapse of moments, by the multiplicity of events which have been crowded into them.

"We all know that our estimate of time depends on the number of circumstances which successively attract our notice. When an event occurs which powerfully impresses the mind, we watch every one of the minutest changes that take place, and the time which elapses before the whole event is completed appears to be proportionally prolonged. Thus we hear of earthquakes in which the commotion of the earth is said to have continued during the space of eight or ten minutes, although in all probability they lasted for no longer time than thirty seconds; and in the same manner we may account for the mistakes to which I have just alluded. When the infidel sultan of Egypt refused to believe that Mohammed could have ascended into the seven heavens, and held one thousand and one conferences with the Deity in the brief space of a few minutes, the Mussulman divine, who was consulted on the occasion, endeavoured to bring his majesty to a more strict faith, by demonstrating that a short space of time was converted into a long one when a great number of important events were crowded into it." (p. 93.)

The treatment of the drowned is similar to that of the strangled. If the removal from the water take place before the diaphragm has ceased to act, respiration may be resumed naturally; if not, artificial inflation is to be employed. And at two periods such inflation may be necessary; first, during the short interval between the cessation of the natural efforts to respire and the cessation of the heart's action; second, when the patient lies in a state of stupor, in consequence of the injurious effects produced by transmission of dark coloured blood to the brain. During resuscitation, "it must be of importance to supply the waste of animal heat, by placing the patient in a warm temperature;" the warm bath forms a simple and convenient method of attaining this object; and it may produce another good effect, "by promoting the natural efforts to respire." Abstraction of blood is of even more doubtful propriety than in cases of strangulation. And there are other things which should not be done.

"We have been directed to employ friction of the surface of the body for the purpose of assisting the circulation of the blood; as if this could do any real good when the action of the heart is ceased; or as if it would not do actual harm by overloading (if I may be allowed to use such an expression) the right auricle and ventricle, when the action of the heart was still going on. The injection of tobacco, and the application of stimulants, belong to the same class of remedies which are either mischievous or useless, proposed formerly by those who did not know what to do, but who thought that they were expected to do something, but now rejected by a more enlightened physiology." (p. 99.)

In the same lecture, a few observations are made on "*death from lightning.*" The substance of them is found at p. 104.

"It appears to me that the facts which I have been able to collect relating to this subject lead to this conclusion, that the influence of lightning, or of a powerful shock of electricity, in the majority of cases, is expended chiefly in disturbing, or destroying, the functions of the brain; and the treatment necessary to counteract the effects of the injury may be comprised in a few words.

"Expose the body to a moderate warmth, so as to prevent the loss of animal heat, to which it is always liable where the functions of the brain are suspended or impaired, and inflate the lungs, so as to imitate natural respiration as nearly as possible, whenever the animal breathes with labour or difficulty, or when he has ceased to breathe altogether by his own efforts." (p. 104.)

LECTURE V contains an account of "*some cases of cysts containing watery fluid, apparently connected with the liver*"—prominent fluctuating tumours formed in the hypochondriac region, with more or less distress. Tapping was had recourse to, and clear, colourless, watery fluid escaped; not coagulable by heat. In one case a fatal result almost took place, in consequence of suppuration having occurred. The cyst inflamed; the neighbouring parts became matted together; by ulceration the suppurated cyst made its way into the intestinal canal, and was thence discharged. The supposed reason of the untoward inflammatory attack is thus stated:

"I suspect that in this instance I made an error in being over-anxious to draw off every portion of the fluid, and that in compressing the cyst for this purpose the canula of the trocar became a source of irritation, and laid the foundation of the inflammation which followed. I have seen the same thing happen in more than one case of ovarian dropsy, where the over-anxiety of the surgeon to empty the tumour completely has been followed by inflammation of the ovarian cyst, and placed the patient's life in danger." (p. 112.)

LECTURE VI, treats of "*ununited fractures.*" Of this chapter there is little or nothing requiring notice or comment, until we come to page 126, and there we find a good description of the state of parts in the ununited fracture. Usually, the two fragments are connected by a ligamentous substance; firm and dense, but not of a distinctly fibrous structure. In some cases, however, a new joint is ready formed. And as, on this point, our author speaks more decidedly, than is usual, as to cartilaginous tipping of the ends of the bones, and production of new synovial membrane, we had better let him speak for himself.

"A new joint is formed—absolutely a false joint. The broken ends of the bone become rounded: there is a capsule nearly as thick as the capsule of the hip-joint or shoulder; this capsule is fibrous, like ligament; it is attached to the bones above and below the fracture, and there is a cavity, like the cavity of a joint, in which the broken ends of the bone lie. And there is more than this: the rounded ends of the bone being covered by a thin, ligamentous substance, and the inner surface of capsule being lined by a smooth membrane, like the synovial membrane, and capable not only of secreting synovia, but of secreting it in abundance. The capsule is a new formation; and the synovial membrane is a new formation also. It is not to be wondered that a synovial membrane should be formed under these circumstances. It seems very easy for the system to construct a membrane of this description. The bursæ mucosæ are made of synovial membrane, just like that of the joints. There is a bursa between the patella and the skin, and this in housemaids sometimes becomes diseased, and converted into a hard lump or tumour. I have frequently removed such a tumour from the knee of a housemaid, and some time afterwards, on examining the limb, I have been satisfied that the bursa had been regenerated. Nor is this a mere supposition; I have positive proof that the fact is as I have stated it. There was a woman in the hospital from whose knee the late Mr. Rose removed an enlarged bursa. A year or two afterwards she returned, and came under my care; and not only had the bursa been regenerated, but the new one had become diseased like the old one, and I had to repeat the operation which Mr. Rose had performed formerly. She had gone back to her former occupation, which included a good deal of kneeling, and, under the influence of pressure, the new bursa had become converted into the same diseased structure as the old one. These cases of artificial joints are comparatively rare; the union by ligamentous substance is much more common." (pp. 126-8.)

As to treatment, Sir Benjamin prefers what he terms "Mr. Amesbury's method;" keeping the ends of the bones in perfect repose, and at the same time applying pressure, particularly on the broken surfaces, so as to keep them in the closest possible contact with each other. This may certainly lay fair claim to our recommendation; "if it do no good, it can do no harm." For ourselves, we have quite made up our minds that the most feasible mode of treatment is that by subcutaneous puncture; as first proposed, we believe, by Professor Miller of Edinburgh:* this converts the parts into a close resemblance of the state immediately consequent on the infliction of the original fracture, and obtains, in consequence, union by satisfactory callus.

Lecture VII. "*On sero-cystic tumours of the female breast.*" This form of tumour, mainly through the services of Sir B. Brodie, has been long well known to the public. Notwithstanding, its peculiarities may be here briefly restated. In its first stage, it consists of one or more membranous cysts; probably produced by dilatation of portions of the lactiferous tubes. The swelling is globular, painless, moveable, and imbedded in the substance of the gland. The contained fluid is straw-coloured and transparent. The general health is unaffected. The ordinary age is between puberty and the middle period of life. By puncturing the cyst and withdrawing the fluid, temporary subsidence of the tumour is obtained; but the contents, in no long time, reaccumulate. To prevent reproduction, our author employs a stimulant embrocation to the skin; but, avowedly, with a result by no means invariably successful. Probably it were better to treat these, as other serous cysts of adventitious growth or enlargement, by injection of iodine. But should any of our readers prefer the more gentle mode, here is the embrocation: "℞ *spiritûs camphorati, spiritûs tenuioris,* āā· ℥iiiss; *liquoris plumbi diacetatis,* ℥j; *fiat embrocatio.*" (p. 152.)

In the second stage, the contents of the tumour thicken and grow opaque; the cysts take on a productive action, studding their inner surfaces with "growths or excrescences," and at the same time, it is probable, deposit of adventitious matter takes place on the exterior, constituting a stroma, as it were, in which the cysts come to be imbedded. Sometimes the cysts become wholly filled with solid matter; the originally cystic structure only becoming apparent when a section has been made of the compact mass.

In the third stage, the integument is involved and ulcerates; or an aperture is made by wound or accident. The contents then protrude, in a fungous form, and both structure and action are prone to degenerate.

* Principles of Surgery, p. 602.

It is obvious that the only satisfactory treatment of the last two stages, is early and free removal by the knife. Though simple or "benign" at first, this tumour, like all other cystic formations, is apt to change sadly for the worse; more especially when the open condition has succeeded to the occult.

LECTURES VIII and IX treat of "*varicose veins and ulcers of the legs.*" The disease is well described; but nothing new is advanced, and nothing requires special notice. As to treatment, our author arrives at the conclusion, with which we feel pretty certain that the great majority of the profession fully agree, namely, that in ordinary cases the palliative treatment is enough, and that the means of obtaining radical cure should be reserved only for those cases which are severe—by extent of varix, by amount of pain, by repeated inflammatory accessions, by ulceration, or by hemorrhage. Of the various methods of radical cure he seems to have no decided preference; at least he hesitates between two:—his own method by subcutaneous section, and that of Velpeau by the twisted suture. For ourselves, we have no hesitation in awarding the palm of superiority to the latter; as equally safe, and rendering obliteration of the veins much more certain. Whatever mode be followed, however, careful bandaging must be employed for a long time afterwards; as there is certainly a strong tendency, otherwise, to the establishment of fresh varices at other parts of the limb.

LECTURE X is a valuable discourse "*on the cases of scirrhous tumours of the breast which require an operation.*" An opinion on a disputed, or at least uncertain point, is given by a thoroughly and extensively practical man; and that opinion coincides with what is understood to be the general feeling of the profession.

"You may divide scirrhous tumours of the breast into two classes: one where there is a conversion of the gland of the breast itself into the scirrhous structure, there being no well-defined margin to it; the other, where there is a scirrhous tumour imbedded in what appears to be otherwise a healthy breast, as if it were altogether a new growth, there being a well-defined boundary to it.

"In the first order of cases, where the tumour has no distinct boundary, and where there is a conversion of the gland of the breast into the diseased structure, the operation not only never succeeds in making a permanent cure, but it rather hastens the progress of the disease. The patient dies within two or three years, and probably much sooner, from an effusion of fluid into the cavity of the pleura." (pp. 195-6.)

The following circumstances are to be considered as excluding any successful result of operation: 1. Involvement of the skin; by scirrhous tubercle formed in its substance; by conversion of the tissue into a thickened and brawny state; or by a dimpled tying down of part of the skin, by prolongations of the tumour which at these points cause incorporation. 2. Much retraction of the nipple. 3. Contamination of the glands in the axilla. 4. Adhesion of the tumour to the parts beneath.

On the other hand, under the following circumstances, the operation may be had recourse to, with a fair prospect of a fortunate issue:

"Where on a careful examination no appearance of disease can be detected in the skin; where there is no dimple in the skin over the tumour; where there is no diseased gland in the axilla; where there is no sign of internal mischief; where there is no adhesion of the breast to the parts below; and where the patient is not very much advanced in life;—in a case where this fortunate combination of circumstances exists, we may presume that there is a reasonable chance of an operation being successful." (p. 199.)

Further, Sir Benjamin coincides with the ordinary opinion, that in certain hopeless cases, it may be our duty to operate; without any hope of ultimate cure, but merely in order to palliate suffering, and perhaps protract existence. Even then, however, rejecting "altogether those cases in which the skin is distinctly contaminated by the disease, whether it be that there are scirrhous tubercles in it, or that it be converted into the brawny structure which I have formerly described. In neither of these cases will the patient obtain even a respite by submitting to an operation." (p. 206.) Tumours which originate in the nipple are supposed to offer a better chance of cure, by operation, than those which commence in the substance of the gland. On this point, let the reader consult Dr. Walshe's treatise.

LECTURE XI, treats of "*corns and bunions;*" but seems rather out of place, and might have been omitted without much loss.

LECTURE XII. "*On the administration of mercury in cases of syphilis.*" Our first remark here is, that the author, in our humble opinion, leans rather much to the mercurial mode of treatment; that is to say, he seems to express himself so as to lead the unwary to believe that no case of syphilis can be cured, or well cured, without the use of mercury; and, further, that the employment of mercury will seldom if ever prove thoroughly beneficial, unless given in large quantity, and for a long period. We need not remind our readers that to the assigning of such a broad position to the antisyphilitic mineral, a large body of the profession stoutly and wisely demur. And they do not agree with Sir Benjamin in thinking that they have retrograded in their art, by departing from the Pearsonic treatment of syphilis. As to the mode of exhibition, our author is very partial to inunction.

"If you ask me which is the best way of using mercury where the symptoms of syphilis are not of the very mildest character, I must say that that by inunction is infinitely to be preferred. Mercurial inunction is dirty, laborious, and troublesome, and it makes the matter public to the family in which the patient lives; for one or other of these reasons it will generally be unpleasant to him. But it has these advantages: it is much less liable to gripe or purge; it cures the disease a great deal better, and does not damage the constitution half so much, as mercury taken by the mouth; nay, I will go so far as to say, that, except in the slighter forms of the disease, you really cannot depend upon any other kind of mercurial treatment for the production of a cure. You may patch up the disease by giving the remedy internally, but it will return over and over again, and then you may cure it at last by a course of mercurial ointment properly rubbed in. I say properly rubbed in, for much depends on this. The patient, if not well instructed, will perhaps continue the friction for a few minutes; but it ought to be continued before a fire at first for at least half an hour, and very frequently for three quarters of an hour. After some time the ointment will be absorbed more readily, and it may then be rubbed in for a shorter period." (pp. 241-2.)

To children, especially he conceives inunction applicable. And the following is his mode of using it:

"I have provided a flannel roller, on one end of which I have spread some mercurial ointment,—say a drachm, or more; and I have applied the roller, thus prepared, not very tight, round the knee; repeating the application daily. The motions of the child produce the necessary friction; and the cuticle being thin the mercury easily enters the system. This causes neither griping nor purging; in a child it does not even in general cause soreness of the gums; but it cures the disease. Very few of those children ultimately recover in whom the mercury has been given internally; but I have not seen a single case in which this other method of treatment has failed." (pp. 244-5.)

We cannot take leave of Sir Benjamin, in this chapter, without thanking him very heartily for "putting in a good word" for sarsaparilla; a useful medicine, which it is too much the fashion of the present day to neglect and decry.

LECTURE XIII. "*On tic-douloureux, or facial neuralgia.*" The uncertainty of our pathology of this lamentable ailment is well stated; in regard to those cases in which the mischief obviously depends on some disorder of the system at large, not on any local affection.

"There is something or another, somewhere or another, in the system, which acts as a source of irritation to the nerves of the face; but where that something is, and what it is, we cannot discover." (p. 259.)

Contrary to what we apprehend is the general impression, Sir Benjamin maintains that little or no good ever follows the removal of diseased teeth.

"I never knew a case in which a patient was relieved of a genuine *tic douloureux* by the extraction of a tooth; and I remember in a conversation which I had with an experienced dentist some years ago, that he told me that he had very frequently been called upon to extract teeth on these occasions, and that he was not aware that the operation had been of service in any one instance." (p. 256.)

It may be so; yet we should consider it but a clumsy practice which would permit diseased and useless teeth to remain in the head of a patient afflicted with tic douloureux of the face.

As to the merits of the "*operation*" for tic, no one will dispute the propriety of its being placed in the following position.

"It is altogether an unscientific operation, from which we have no more right to expect benefit than we should have from the amputation of the testicle in a case of pain referred to that organ in consequence of a calculus being lodged in the ureter." (p. 261.)

In speaking of the empirical use of certain remedies, our author states that he has never known any benefit to accrue from increasing the dose of the carbonate of iron beyond a drachm thrice daily, and properly insists on the occasional administration of purgatives, to prevent accumulation of the medicine in the colon, as is otherwise apt to occur. But in regard to these, and all other *quasi specifics,* it is most important to remember that it by no means follows that because they are doing no good they are doing no harm. "*No one can be dosed constantly with medicine without the health being ultimately injured by it; and if you have not some reasonable grounds for giving medicine, you have no right to run the risk of doing harm by its continued exhibition.*" (p. 265.) We are satisfied that many systems have been improperly ruined in empirical and unsuccessful attempts to purchase a local advantage at too high a cost. How often in amaurosis, for example, has not the routine of mercury, colchicum, strychnia, bleeding, blistering, left the patient much worse than it found him? As regards *new remedies,* the following quotation contains a wholesome truth:

"I shall take this opportunity of observing, that I am not disposed to try indiscriminately all the new remedies which in these days are being constantly brought before society; nor can I think well of this modern fashion of resorting on all occasions to novel methods of treatment. I advise you, if you wish to succeed in your profession, and to be useful to society, to pursue a different course. Make yourselves masters of the old remedies. Learn how to handle them, and what good they will do, and, as a general rule, have recourse to them in the first instance. If the old remedies fail, and you are at a loss as to what you should do, then, and not till then, have recourse to the new ones. If you always begin with new remedies, you throw away the valuable results, not only of your own experience, but of the experience of those who have gone before you. You have to begin, as it were, *de novo;* and the first consequence of this will be, that you will not cure your patients; and the second, that you will have none to cure." (pp. 267-8.)

Lecture XIV. "*On fatty or adipose tumours.*" In some cases of lipoma, a hypertrophied condition of the integument and subcutaneous adipose tissue—not an example of the true fatty tumour—our author is of opinion that benefit is to be derived from the internal use of liquor potassæ in large doses; a drachm, thrice daily in small beer.

The next six chapters treat of *mortification.* They are, for the most part, quite elementary in their character; but the following points are more suited for especial notice:

"Another plan may be adopted to prevent mortification from pressure; that is, to prevent the inflammation which precedes it. The thicker the cuticle the more it will protect the parts beneath; you may, if you attend to it in time, add to the thickness of the cuticle by stimulating the surface of the skin. Nurses know this very well, for when patients are bed-ridden they wash the parts subjected to pressure with brandy. What is still better is a lotion composed of two grains of bichloride of mercury to an ounce of proof spirits. When you think that a patient is likely to be confined so long in bed that sloughs may be formed on the os sacrum, begin at an early period to wash the parts two or three times a day with this lotion. I have found it useful in other cases where a patient suffers from pressure. For example, in a case of hernia, which requires to be supported by a very powerful truss; the truss galls and frets the skin, and may at last cause inflammation and sloughing; but under the use of a stimulating lotion a thicker cuticle is generated, and such mischief is avoided." (pp. 311-12.)

"Almost always, when you use a caustic, it is prudent to have some counteragent at hand to stop its action if it reaches a sound part. Acids may be neutralized by alkalies; caustic potash may be neutralized by vinegar, or by a solution of the diacetate of lead. If you are afraid of nitrate of silver burning the neighbouring parts, its action may be neutralized by common olive oil. A solution of bicarbonate of potash will decompose chloride of zinc, and so with other caustics." (pp. 328-9.)

Subcutaneous nævi may be destroyed by the subcutaneous puncture of a knife, freely lacerating the morbid structure, followed by a probe dipped into nitrate of silver, melted in a platina or silver spoon. The advantage of such a procedure is, that when the nævus is situate on the face, it may be removed effectually thus, without having any cicatrix or other deformity. The operation "causes inflammation and sloughing, at the same time obliterating the vessels beyond the margin of the slough."

Is our author certain that the following is a fact?

"The fact is, that a wound always heals much more readily after the application of caustic, than after the use of the knife. Take two cases: if you destroy one tumour of a given size by the knife, and the other, supposed to be of the same size, by caustic, in spite of the time occupied by the separation of the slough, the sore in the latter case will be healed sooner than that in the former." (p. 334.)

We think we could get up a good deal of evidence to the contrary.

In applying caustics to the scalp, observe caution. Extensive destruction of the pericranium may cause necrosis of the corresponding portion of the skull, throughout its entire thickness; this is likely to be associated with purulent detachment of the dura mater, and from that a train of fatal symptoms may arise. Our author has seen this unfortunate sequence.

Chloride of zinc is not a suitable escharotic for soft parts, unless a raw surface previously exist. When applied to the unbroken skin it produces intense pain, and fails of the escharotic result.

Most cordially do we agree with the following. There are a few cases certainly, which we need not here stop to specify, in which the actual cautery is preferable to caustics; but only a few. Of late years we were much afraid that a retrograding tide had set in in favour of the "rude piece of farriery;" but for some time back we have been charmed to find such fears gradually subsiding, the cauteries having again resumed their little disturbed position in the armamentarium.

"The actual cautery does nothing which caustics would not do as well or better, and it is much more alarming and frightful, both to the patient and to bystanders. It was the habit of surgeons here, fifty or sixty years ago, to use the actual cautery to a great extent; and it appears to be one of the many proofs of the advancement of English surgery that we have got rid of what Sir Astley Cooper used to call 'a rude piece of farriery.' " (p. 345.)

LECTURE XIX, "*On senile gangrene,*" we could have wished expunged. The description contains nothing new, and some of the treatment is, in our humble opinion, both old and bad. Our readers need not to be told that the animal-food and port-wine system is not that which, in a large proportion of such cases, ought to be recommended.

LECTURE XXI.—"*On chronic abscess of the tibia.*" Here Sir Benjamin is peculiarly at home. The profession is undeniably indebted to him for a large boon, in having determined the existence of this formidable disease, and the simple and satisfactory means whereby it may be remedied. The affection is most liable to occur in the tibia, at either extremity, but most frequently at the upper. In 1824, the first case occurred to our author; but unfortunately its true nature was not discovered till after amputation. In 1827, a second patient appeared; and, being subjected to the suitable treatment, retained his limb, and was rid only of the disease. Since then it must indeed be very satisfactory to Sir Benjamin

"to know that this simple method of treatment has already preserved many limbs which must have been sacrificed otherwise, and it cannot be doubted that it will be the means of preserving many more, when it is generally adopted by those who are engaged in operative surgery." (p. 403.)

A trephine of less size than what is ordinarily used for the skull is generally sufficient; and it should have no projecting rim or shoulder, so as to permit of deep penetration. The circumstances which point to the operation are the following:

"When the tibia is enlarged from a deposit of bone externally—when there is excessive pain, such as may be supposed to depend on extreme tension, the pain being aggravated at intervals, and these symptoms continue and become still further aggravated, not yielding to medicines, or other treatment that may be had recourse to, then you may reasonably suspect the existence of abscess in the centre of the bone. You are not to suppose that there is no abscess because the pain is not constant; on the contrary, it very often comes on only at intervals, and in one of the cases which I have related there was, as I then mentioned, an actual intermission of seven or eight months. After the disease has existed a certain number of years, indeed, the pain never entirely subsides, but still it varies, and there are always periods of abatement and of exacerbation." (p. 404.)

Our last quotation is from the preface.

"If what I now offer to their perusal should prove acceptable to the profession, I may venture to publish a Second Series of my Lectures, as soon as I find the necessary leisure for that purpose." (p. iii.)

We venture to say that the profession will gladly take Sir Benjamin at his word. But if we may be allowed, we would throw out a hint for his consideration in regard to the next volume; namely, the propriety of not giving us the lectures exactly as they were delivered; but holding back the elementary, the well-known, and—even though good—the stale,—giving prominence and bulk to his own views and practice when varying from the ordinary routine, and bringing all up quite to the level of modern scientific advancement.

---

